# How do small quantities of cartilage sodium channels play a significant role in osteoarthritis?

**DOI:** 10.1002/ctm2.1634

**Published:** 2024-03-26

**Authors:** Xiaohong Kong, Chuan‐Ju Liu

**Affiliations:** ^1^ Department of Orthopaedics and Rehabilitation Yale University School of Medicine New Haven Connecticut USA

**Keywords:** cartilage chondrocytes, osteoarthritis, sodium channel Nav1.7, therapeutic target

## 1 INTRODUCTION

Osteoarthritis (OA) is a chronic degenerative joint disease affecting the entire joint, causing pain, stiffness and limitations in mobility and posing a significant burden on global health and the quality of life for millions worldwide.^1^ Despite its prevalence, treatment options remain limited, often focused on symptom management rather than addressing the underlying mechanisms driving joint degradation.^2^, ^3^ OA is characterized by the loss of cartilage, prompting most efforts to develop disease‐modifying treatments to concentrate on molecular events within the cartilage. Our research has centred around the regulation of cartilage homeostasis and OA, with a specific focus on cartilage‐degrading matrix‐associated a disintegrin and metalloproteinase with thrombospondin type I motifs^2^ and inflammatory mediators, particularly tumour necrosis factor receptor signalling.^4^, ^5^, ^6^, ^7^, ^8^ In a recent report, our research revealed the presence and functionality of the sodium channel Na_v_1.7 (encoded by *SCN9A*) in cartilage cells, namely chondrocytes, highlighting Na_v_ 1.7 as a novel therapeutic target for OA.^9^

### 1.1 The key findings and significance

Sodium channels are commonly highly expressed on excitable cells, such as neurons, muscle cells and cardiac myocytes.^10^ In an unbiased genetic screen for identifying the OA‐associated molecules, Na_v_1.7 was, unexpectedly, found to be present and elevated in human OA chondrocytes.^9^ This intriguing and unexpected discovery that chondrocytes express functional Na_v_1.7 was validated through multiple assays, particularly a spectrum of electrophysiological and pharmacological methods. Subsequent serial genetic ablation of Na_v_1.7 in multiple mouse models demonstrated that dorsal root ganglion neuron‐expressed Na_v_1.7 is involved in pain perception, whereas chondrocyte‐expressed Na_v_1.7 governs OA progression as judged by both behaviour and anatomical methods. Additionally, pharmacological blockade of Nav1.7 with selective or clinically used pan‐Nav blockers can simultaneously attenuate the progression of OA and alleviate OA pain.^9^ Mechanistically, Na_v_1.7 deletion or blockade regulates chondrocytes biology and OA through enhancing HSP70 and midkine secretion.^9^

These findings demonstrate that in addition to controlling pain signalling in sensory neurons, Na_v_1.7 within chondrocytes plays a pivotal role in the progression of joint damage in OA. These findings not only better our understanding of the ion channel physiology as well as chondrocyte biology but also provide a basis for the development of Na_v_1.7 blockers as disease‐modifying drugs for treating OA pathologically and symptomatically, thereby expanding appreciation of their clinical utility beyond that of pain killers. Na_v_1.7 opens a rich landscape of exploration and further investigation into the intricate mechanism governing chondrocyte function and cartilage homeostasis, providing fertile ground for translational research and drug development efforts.

### 1.2 The challenges and future direction

While recording sodium channel currents in chondrocytes poses significant challenges compared to neurons, our successful observations reveal the existence of functional Na_v_1.7 channels in human OA chondrocytes. These channels exhibit densities of 0.1–0.15 channels/μm^2^, with channel numbers ranging from 350 to 525 per cell—several orders of magnitude lower than in neurons.^9^ Patch‐clamp recordings indicate that Na_v_1.7 is expressed in approximately 17% of OA chondrocytes.^9^ The key questions arising are why and how such a small number of Na_v_1.7 channels in chondrocytes play a substantial role in OA progression. Unravelling the intricate molecular mechanisms underlying this phenomenon warrants further investigation.

**FIGURE 1:**
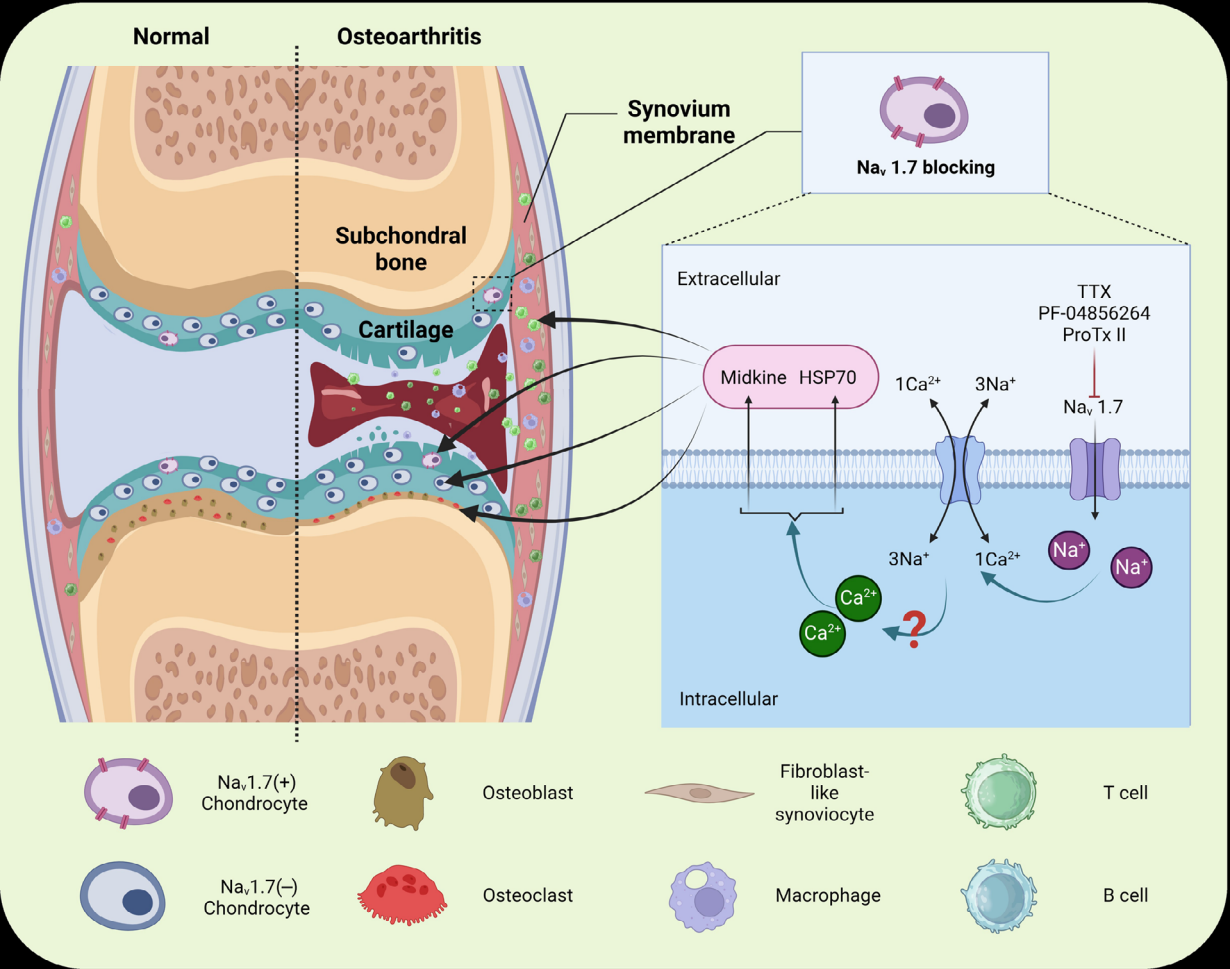
Na_v_1.7 blockade protection against osteoarthritis (OA) through the enhanced release of HSP70 and midkine in an intracellular Ca^2+^ signalling dependent manner. The schematic diagram highlights the increased section of HSP70 and midkine‐regulated chondrocytes biology and the progression of OA via autocrine and paracrine effects. Additionally, the cellular communication among Na_v_1.7 expressing chondrocytes and other joint cells such as synovial fibroblasts and osteoclasts, as well as infiltrating immune cells is the future research direction. We created a figure using biorender.com.

In chondrocytes expressing Nav1.7, the blockade of Na_v_1.7 triggers increased secretion of HSP70 and midkine, which is crucial for Na_v_1.7 blockade‐mediated regulation of chondrocyte biology, in turn impacting joint structure and pain in OA.^9^ The increased release of HSP70 and midkine upon Na_v_1.7 blockade of Na_v_1.7 expressing chondrocytes is possibly the tip of the iceberg. One plausible scenario is that this heightened secretion occurs through autocrine and paracrine effects, influencing neighboring chondrocytes lacking Na_v_1.7 expression, in addition to directly affecting Na_v_1.7‐expressing chondrocytes. This multiplicative effect contributes to the co‐regulation of anabolic and catabolic processes, ultimately influencing the progression of OA (Figure 1).

Considering that Na_v_1.7 blockers influence intracellular Ca^2+^ signalling, thereby impacting the secretion of HSP70 and midkine from chondrocytes, delving into the molecular mechanisms underlying Na_v_1.7‐mediated chondrocyte regulation necessitates a focus on identifying specific mediators within and/or beyond intracellular calcium changes caused by Na_v_1.7 modulation. It is also important to investigate whether calcium alterations, after Na_v_1.7 deletion or blockade, act as the sole second messenger or work in conjunction with other mediators to regulate chondrocyte biology. Additionally, it is worthwhile to explore whether Na_v_1.7 modulation‐induced changes in calcium levels are associated with osteoclast activity and the balance between osteoblasts and osteoclasts. Further investigations should also address the signalling pathways, specifically isolating the molecules linking Na_v_1.7 blockade and intracellular calcium changes, as well as their impact on chondrocyte biology, particularly chondrocyte secretomes.

Given the regulatory roles of HSP70 and midkine in inflammation and chondrocyte proliferation, it is plausible that Na_v_1.7‐positive chondrocytes engage in interactions with surrounding tissues, such as synovial membranes and subchondral bone, by influencing the release of these proteins into the synovial fluid. Therefore, future research should also delve into the cross‐talk and communication pathways among sodium channels Na_v_1.7‐expressing chondrocytes and other joint cells, including synovial fibroblasts and osteoblasts, as well as immune cells infiltrating the joints (Figure 1). Additionally, investigating the interactions of Na_v_1.7 with other membrane‐associated proteins/receptors and lipids that may also play a role in the pathogenesis of osteoarthritis should be considered.

From potential translational and clinical perspectives, exploring combination therapies that target sodium channels Na_v_1.7, along with cutting‐edge approaches, holds promise for enhancing the efficacy of OA treatment. Beyond conventional small‐molecule drugs targeting ion channels, alternative methods such as small‐interfering RNA, antisense oligonucleotides, antibodies and mRNA vaccines provide diverse avenues for OA treatment and prevention. Customizing intra‐articular injections to specific OA tissues may optimize drug bioavailability while minimizing systemic side effects. Additionally, incorporating biomaterials like nanomaterials, which facilitate cartilage penetration and hydrogels, could enhance control over drug delivery. These strategies aim to achieve targeted and prolonged drug release, potentially reducing side effects, lowering dosage frequency and increasing treatment durability.

## 2 CONCLUSION

The discovery of Na_v_1.7 as a chondrocyte regulator and therapeutic target represents a significant milestone in OA research and clinical translation and application in the future. However, we fully acknowledge that Na_v_1.7 regulation of chondrocyte biology and OA pathogenesis is far more sophisticated than currently appreciated. Definitive knowledge will emerge through advanced studies, including the CRISPR/Cas9 technology to genetically modify transcribed and cis‐acting regions of Na_v_1.7, its targets, mediators and co‐factors. As we mentioned, the challenges lie ahead. The collective efforts of clinicians, researchers and pharmaceuticals hold the promise of translating Na_v_1.7 targeting into tangible clinical benefits, ultimately reshaping the landscape of OA management for generations to come. There is still a long way to go to understand thoroughly the molecular mechanism of Na_v_1.7 channels in OA pathogenesis and its clinical application. Nevertheless, we are working towards a future where osteoarthritis is more manageable, letting people have healthier and happier lives.

## AUTHOR CONTRIBUTIONS

XK wrote the manuscript. CL edited the manuscript. All authors contributed to the article and approved the submitted version.

## CONFLICT OF INTEREST STATEMENT

The authors declare no conflict of interest.

## ETHICS STATEMENT

Not applicable
